# Solution Structure of the LIM-Homeodomain Transcription Factor Complex Lhx3/Ldb1 and the Effects of a Pituitary Mutation on Key Lhx3 Interactions

**DOI:** 10.1371/journal.pone.0040719

**Published:** 2012-07-25

**Authors:** Mugdha Bhati, Christopher Lee, Morgan S. Gadd, Cy M. Jeffries, Ann Kwan, Andrew E. Whitten, Jill Trewhella, Joel P. Mackay, Jacqueline M. Matthews

**Affiliations:** School of Molecular Bioscience, University of Sydney, Sydney, New South Wales, Australia; University of Canterbury, New Zealand

## Abstract

Lhx3 is a LIM-homeodomain (LIM-HD) transcription factor that regulates neural cell subtype specification and pituitary development in vertebrates, and mutations in this protein cause combined pituitary hormone deficiency syndrome (CPHDS). The recently published structures of Lhx3 in complex with each of two key protein partners, Isl1 and Ldb1, provide an opportunity to understand the effect of mutations and posttranslational modifications on key protein-protein interactions. Here, we use small-angle X-ray scattering of an Ldb1-Lhx3 complex to confirm that in solution the protein is well represented by our previously determined NMR structure as an ensemble of conformers each comprising two well-defined halves (each made up of LIM domain from Lhx3 and the corresponding binding motif in Ldb1) with some flexibility between the two halves. NMR analysis of an Lhx3 mutant that causes CPHDS, Lhx3(Y114C), shows that the mutation does not alter the zinc-ligation properties of Lhx3, but appears to cause a structural rearrangement of the hydrophobic core of the LIM2 domain of Lhx3 that destabilises the domain and/or reduces the affinity of Lhx3 for both Ldb1 and Isl1. Thus the mutation would affect the formation of Lhx3-containing transcription factor complexes, particularly in the pituitary gland where these complexes are required for the production of multiple pituitary cell types and hormones.

## Introduction

Lhx3 (LIM homeobox protein 3) is essential for specification of many pituitary and neural cell types [1], [2], [3]. Humans that carry mutations in Lhx3 present with combined pituitary hormone deficiency syndrome (CPHDS) [4], [5], [6], [7], [8]. Depending on the site of mutation, affected patients can also exhibit hearing loss and skeletal malformations of the upper body [8], [9].

**Figure 1:**
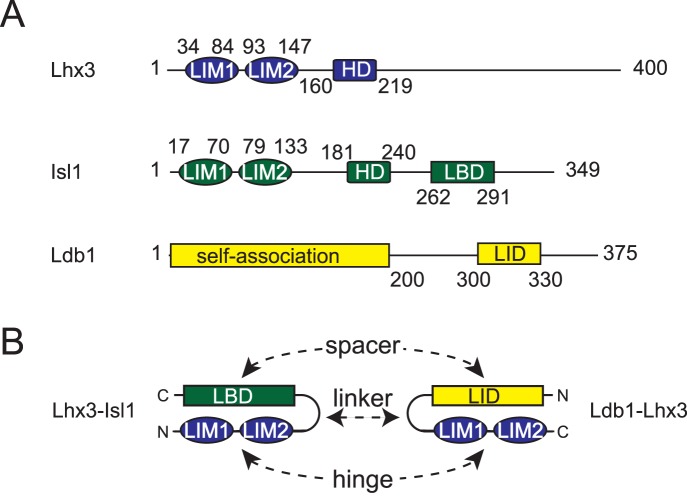
Schematics of Lhx3, Isl1 and Ldb1 proteins and tethered complexes. (**A**) Schematics of mouse proteins as indicated. Numbers refer to the mouse proteins with the domain boundaries as defined in SWISS-PROT. (**B**) Arrangement of domains in the tethered complexes Lhx3-Isl1 and Ldb1-Lhx3. The N and C termini are indicated. HD, homeodomain; LBD, Lhx3-binding domain; LID, LIM interaction domain; LIM1, N-terminal LIM domain; LIM2, C-terminal LIM domain. The linker and the locations of the hinge (located in residues between the two LIM domains) and spacer (corresponding region in the LBD/LID domains) in each construct are shown.

Lhx3 is from the LIM-homeodomain transcription factor family, members of which contain a pair of closely spaced N-terminal LIM domains followed by a central homeodomain (Figure 1A). The C-terminal portion of LIM-homeodomain proteins is generally poorly characterized but in Lhx3 is reported to contain an activation domain [10]. LIM domains (named for the first three proteins in which the domain was found, Lin-11, Isl1 and Mec-3) are zinc fingers that coordinate two zinc ions and mediate protein-protein interactions [11]. The LIM domains from Lhx3 make well-characterized interactions with Ldb1 (LIM domain binding protein 1) and Isl1 (Islet 1) [2], [12], [13], [14], [15] and have been reported to also bind PIT-1 (pituitary-specific transcription factor 1) [16] and SLB (selective LIM domain binding protein) [17]. The DNA-binding specificity of Lhx3 is context dependent, and varies according to the protein isoform [18], [19], or whether Lhx3 is in acting in concert with a protein partner [2], [20], [21], [22]. Disease-causing mutations or post-translational modifications, including phosphorylation, of the LIM domains are likely to affect the biological activity of Lhx3 by modulating protein-protein interactions and modulating binding to DNA targets [5], [6], [10].

The isolated LIM domains from Lhx3 (Lhx3_LIM1+2_) tend to be insoluble and/or aggregate, but soluble stable “tethered complexes” can be engineered in which the LIM interaction domain of Ldb1 (Ldb1_LID_), or the Lhx3-binding domain from Isl1 (Isl1_LBD_) are fused to Lhx3_LIM1+2_ via a flexible glycine-serine linker (Figure 1B) [23], [24], [25]. These tethered complexes are hereafter referred to as Ldb1-Lhx3 and Lhx3-Isl1 (the order of the names indicates the order of the domains in the complex). Our recently determined structures of Ldb1-Lhx3 and Lhx3-Isl1 show that Ldb1_LID_ and Isl1_LBD_ interact with Lhx3 in an essentially identical manner [13], with the two binding partners forming extended chains and contacting the same sites across both LIM domains of Lhx3. The tethered complexes appeared to have some flexibility in the “hinge” between the LIM domains and the corresponding “spacers” in Ldb1_LID_ and Isl1_LBD_ which lie between the two LIM-binding motifs in each of those domains. The hinge comprises Lhx3_F90_, which shows some variation in backbone angle in different conformers/molecules, and the spacers comprise Ldb1_M310–D318_ and Isl1_H273–Q278_, which assume different overall conformations in the Ldb1 and Isl1 structures (including a short region of disorder in Ldb1_G312–G315_ and an extended structure in one chain of Isl1, but a turn in the other chain) [13]. For Lhx3-Isl1 flexibility at the hinge/spacer is supported by small angle X-ray scattering (SAXS) data [26]. The initial part of this study uses SAXS to further characterize the solution structure of Ldb1-Lhx3.

These structures provide us with the opportunity to interpret the molecular effects of disease-causing mutations and posttranslational modification of the LIM domains of Lhx3. The mutation of tyrosine 111 to cysteine, Y111C, is an inherited point mutation found in the LIM domains of human Lhx3 that is associated with CPHDS [4]. Although the sequences of the LIM domains from Lhx3 are almost identical in mammals, the numbering of the human and mouse proteins differs slightly; numbering for the mouse protein is used herein, with human Y111 corresponding to Y114 in the mouse protein. The affected residue lies adjacent to one of the zinc ligating residues, H115, prompting suggestions that the introduced cysteine sidechain in Y114C might displace H115 as a zinc ligand [5], [6]. Two putative phosphorylation sites, T63 and S71 are located in the first LIM domain of human Lhx3 [10], of these residues only S71 is highly conserved across species. The equivalent residue in the mouse protein, S74, lies adjacent to the binding interface within the Lhx3/Ldb1 and Lhx3/Isl1 complexes.

Here we used SAXS to show that the NMR structure of Ldb1-Lhx3, an ensemble of elongated molecules with small differences in angle between the two LIM modules [13], is a reasonable representation of the solution structure of this tethered complex. We used yeast two-hybrid assays to show that pseudophosphorylation of Lhx3-S74 does not affect binding to Ldb1 or Isl1, but that Lhx3(Y114C) has reduced levels of binding to both key partners. NMR spectroscopy and stability studies demonstrated that Lhx3(Y114C) does not alter the zinc-ligation characteristics of this domain, but does affect the stability and local structure of the second LIM domain of Lhx3.

## Materials and Methods

### Constructs

Unless otherwise specified, residue numbers and sequences refer to mouse proteins Lhx3 (UniProtKB/Swiss-Prot ID P50481-1), Ldb1 ((UniProtKB/Swiss-Prot ID P70662-3) and Isl1 (UniProtKB/Swiss-Prot ID P61372-1). All constructs were cloned into pGBT9 and pGAD10 (Novagen) for yeast two-hybrid experiments, or pGEX-2T (GE Healthcare) for expression in bacteria with an N-terminal GST (glutathione-S-transferase) tag. All plasmids were sequenced to confirm identity (SUPAMAC, Royal Prince Alfred Hospital, Sydney). The Ldb1-Lhx3 tethered construct contains Ldb1 residues 300–339 (Ldb1_LID_), a synthetic linker (GGSGGHMGSGG), and Lhx3 residues 28–153, and were constructed, expressed and purified as described previously [24], [25]. Ldb1-Lhx3(Y114C) was generated by PCR using overlap extension mutagenesis. Protein concentrations were determined by absorbance at 280 nm using theoretical extinction coefficients, ε_280 nm_ of 12,210 M^−1^ cm^−1^ for wildtype Ldb1-Lhx3 and 10,845 M^−1^ cm^−1^ for Ldb1-Lhx3(Y111C).

### Yeast Two-hybrid Assays

pGBT9 and pGAD10 plasmids were co-transformed into AH109 cells (Clontech), as described previously [27]. All selective media lacked leucine (-L) and tryptophan (-W) to ensure co-transformation of bait and prey plasmids was maintained. For screening of interactions, all media were further deficient in histidine (-H) but contained or lacked additional reagents for selection of different affinity interactions. Selective media were either supplemented with 40 μg mL^−1^ X-α-gal (Progen) and 1 mM 3-amino-1,2,4-triazole (3-AT; Sigma) (-L-W-H +3-AT; moderate stringency selection), or were additionally deficient in adenine (-L-W-H-A; high stringency selection).

### Circular Dichroism Spectropolarimetry

Far UV-CD experiments were recorded at 20°C on a Jasco J-720 spectropolarimeter equipped with a Neslab RTE-111 temperature controller. Protein samples (30 μM) were prepared in 20 mM Na_2_HPO_4_, 40 mM NaCl, 1 mM DTT, pH 6.8, placed in a 1-mm path length quartz cell seated in a water-jacketed cell holder. CD spectra were collected over the wavelength range 205–260 nm, a speed of 20 nm/min, 1-nm step resolution, 1-nm bandwidth and a response time of 1 s. Final spectra were the average of five scans, and were buffer baseline corrected. Estimates of secondary structure were determined using CDPro [28].

### Chemical Denaturation Experiments

Protein (2.5 μM) in 20 mM Na_2_HPO_4_, 40 mM NaCl, 1 mM DTT, pH 6.8 and Gdn.HCl as indicated were incubated at 25°C for 2–3 h. Fluorescence emission spectra (320–370 nm) were recorded using a Varian Cary Eclipse fluorescence spectrometer (Palo Alto, CA, USA), with an excitation wavelength of 295 nm. Slit widths were 10 nm, data were collected in 1-nm steps with an averaging time of 0.5 s, and spectra were buffer baseline corrected.

### Nuclear Magnetic Resonance

NMR samples contained 1 mM 2,2-dimethylsilapentane-5-sulfonic acid (DSS) and 10% D_2_O. Spectra were collected at 310 K using a Bruker DRX-600 spectrometer equipped with a 5-mm triple resonance probehead (TXI) and three-axis pulsed field gradients. The spectral widths/carrier frequencies were: ^1^H - 13 ppm/frequency of the water peak; and, ^15^N - 35 ppm/118.3 ppm, or 140 ppm/205 ppm for the detection of histidine sidechains [29].

Data were processed using XWINNMR 3.5 or TOPSPIN 1.2 (Bruker Biospin). Spectral analysis was carried out using SPARKY versions 3.05–3.113 [30]. ^1^H frequencies of all spectra were directly referenced to DSS, and ^15^N frequencies were referenced indirectly [31]. Spectral resolution in the directly detected dimension was enhanced by apodisation with either a Lorentzian-Gaussian window function (LB  = 0.1, GB  =  –3) or a squared sine bell function (shifted by π/2.6 radians) in indirectly detected dimensions. Digital resolution was enhanced by zero-filling (once in each dimension) and linear prediction (in the ^15^N dimension only) before Fourier transformation. Polynomial baseline corrections were applied to processed spectra where appropriate.

### Small Angle X-ray Scattering

Small-angle X-ray scattering data were collected from solutions of Ldb1-Lhx3 (4.5–9 mg mL^−1^) with 20 mM Na_2_HPO_4_, 40 mM NaCl, 1 mM DTT, pH 6.8 and a matched solvent blank at 283 K for 10 s intervals over 30 min using a line-collimated SAXSess scattering instrument (Anton Paar, Graz, Austria) equipped with a CCD detector [32]. Scattering data were reduced to *I*(*q*) vs *q* (where *q*  =  (4πsin*θ*)/λ, 2*θ* is the scattering angle, and λ the X-ray wavelength, CuK_α_, 1.54 Å) using the SAXSQuant 2.0 software package (Anton Paar, Austria) that corrects for sample absorbance and detector sensitivity, and normalises and subtracts solvent from protein+solvent to yield *I*(*q*) *versus q* for the protein alone. The reduced scattering profiles were all placed on an absolute scale using the scattering from water [33]. The programs GIFT [34] and GNOM [35] were used to calculate the probable distribution of atom-pair distances within Ldb1-Lhx3 (*P*(*r*) *versus r* profiles), accounting for the 10-mm slit-geometry of the instrument, from which the radius of gyration (*R_g_*), maximum dimension (*D_max_*) and forward scattering intensity at zero angle (*I*(0)) were extracted; the two programs gave essentially the same results. A molecular weight estimate of Ldb1-Lhx3 was derived from *I*(0) as described in [33] using values for the contrast (Δ*ρ*) and partial specific volume (υ) calculated in CONTRAST from the MULCh program suite [36]. Although MULCh was designed for use in small-angle nuclear scattering (SANS), it can also be used to process SAXS data. An *I*(0) analysis of the 9 mg mL^−1^ sample was indicative of some aggregation and was not further analysed. Guinier analysis of the SAXS data was performed using PRIMUS [37]. *Ab initio* shape restorations of Lhx3-Ldb1 were performed 10 times using the program DAMMIF [38] and a consensus model developed via the spatial alignment and averaging of each solution combined with standard phase-occupancy and volume corrections [39]. Rigid-body modelling against the SAXS data was performed using the high resolution structures of each LIM-half of the complex and the program BUNCH [40]. CRYSOL [41] was used to evaluate the fits against the scattering data of the final BUNCH model and each individual Ldb1-Lhx3 NMR structure derived from the NMR ensemble. CRYSOL was also used to calculate the theoretical scattering profiles of the resultant high-resolution models which were used to derive model *P*(*r*) vs *r* profiles for comparison with the experiment [35].

## Results

### SAXS Data are Consistent with NMR Data for Ldb1-Lhx3

**Figure 2:**
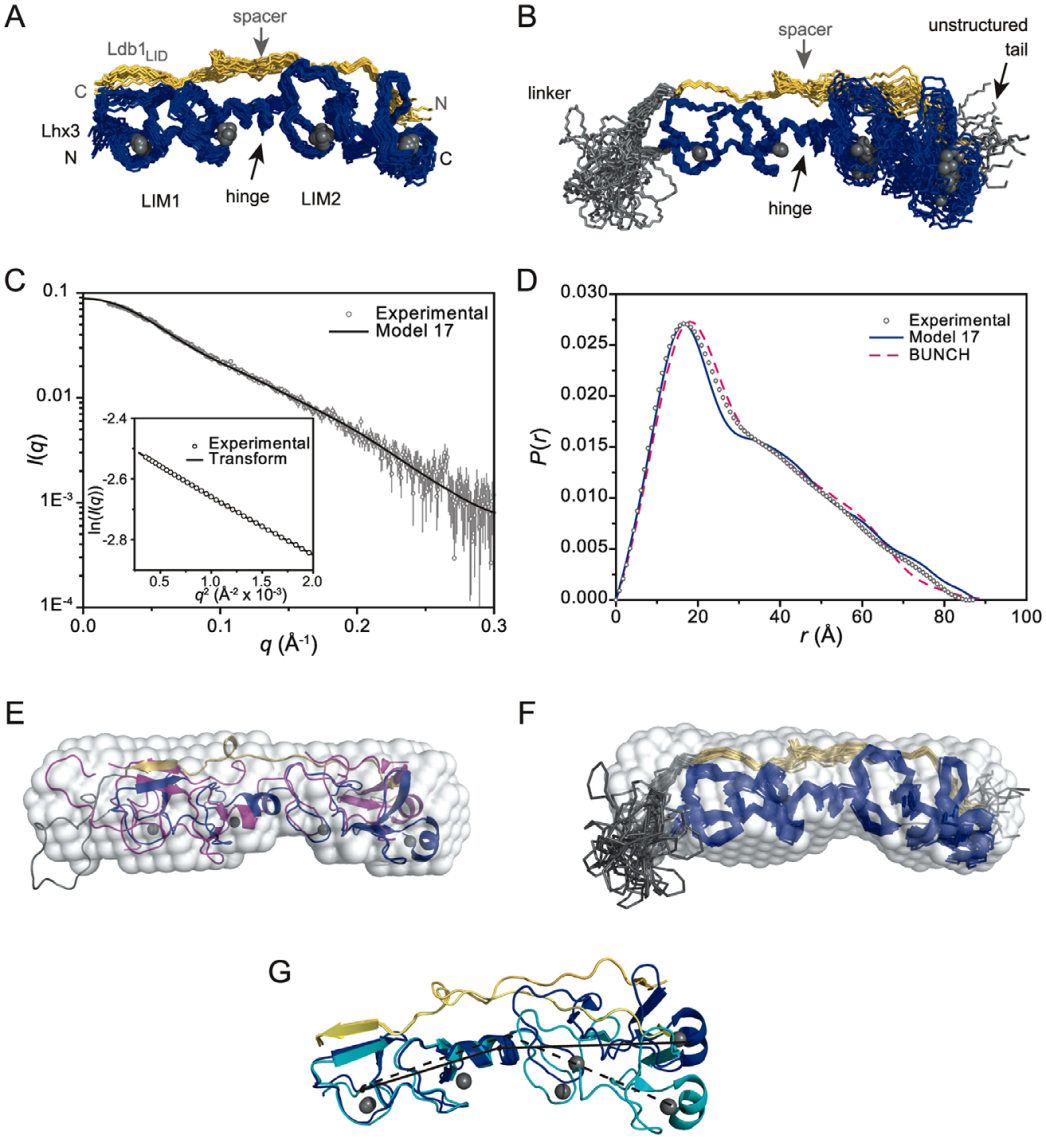
Solution structure of Ldb1-Lhx3. (**A**) NMR structure of Ldb1-Lhx3 (pdb accession code: 2JTN) with Ldb1_LID_ in yellow and Lhx3 in blue; zinc ions are depicted as grey spheres. The 20 lowest energy models are aligned over the backbone atoms of both LIM domains. Only the structured regions are shown. The positions of the N and C termini of the interacting domains of Lhx3 and Ldb1, and the position of the two LIM domains from Lhx3 are indicated. (**B**) Ldb1-Lhx3 with the same 20 conformers from (A) aligned over the backbone atoms of Lhx3_LIM1_ and the corresponding region of Ldb1. The unstructured linker and tail from each model is shown in grey. (**C**) Scattering data for Ldb1-Lhx3 (*grey circles*) shown as *I*(*q*) versus *q* with the corresponding Guinier plot (ln(*I*(*q*)) versus *q* ^2^) in the inset. The fit is for Model 17 from the Ldb1-Lhx3 NMR ensemble as generated by CRYSOL (*black line*). The black line in the inset is the fit to the data generated by GNOM. (**D**) *P*(*r*) profiles from experimental scattering data for Ldb1-Lhx3 (*grey circles*) and calculated scattering profiles from Model 17 of the NMR ensemble (*blue line*) and the generated BUNCH model (*dashed magenta line*). (**E**) Alignment of Model 17 of the Ldb1-Lhx3 NMR ensemble (*coloured as in Panel B*), BUNCH model (*magenta*) and the *ab initio* DAMMIF reconstruction from the scattering data (*transparent white surface*). (**F**) Alignment of the NMR ensemble (*coloured as in Panel B*) with the same DAMMIF consensus model. (**G**) Most disparate models from the 2JTN NMR structure. Model 1 (blue/yellow) has an angle between the Cα atoms of the first zinc-coordinating residue (C34), the hinge residue (F89) and the last zinc coordinating residue (D147) of 163.7° (solid black line). Model 9 (cyan/light yellow) has an angle over the same atoms of 135.1° (dashed black line). Models are aligned using the backbone atoms of the LIM1 domain. Images of structures were created in Pymol.

Our previously determined NMR structure of Ldb1-Lhx3 is elongated, with members of the NMR ensemble comprising two LIM modules (LIM1 and LIM2, each with the contacting region of Ldb1_LID_) but with angles at the “hinge”/”spacer” between the two modules that vary by up to ∼30°, and the tether between Ldb1 and Lhx3 and residues at the C-terminus of the construct are unstructured (Figure 2A and B) [13]. Similar tethered complexes (Lhx3-Isl1 and Lhx4-Isl2) can give rise to extreme angles in crystal structures [13], [26]. Relatively few long distance restraints exist between the two modules in the NMR structure, and residual dipolar coupling constants could not be determined for this complex [13], creating some uncertainty as to whether the gross structure of the Lhx3/Ldb1 complex was adequately described. Thus we used small-angle X-ray scattering (SAXS) to independently define the global conformation of Ldb1-Lhx3 in solution. Analysis of the molecular weight derived from the forward scattering intensity at zero angle combined with the linearity of the Guinier plots in the low-*q* regime indicates that Ldb1-Lhx3 exists as a monodisperse sample of monomers (Table 1; Figure 2C) [42].

**Table 1:** Structural parameters for Ldb1-Lhx3 from small-angle X-ray scattering data.

*D*_max_ (Å)	88
[protein] (mg mL^−1^)	4.50±0.12^a^
*I*(0) (cm^−1^)	0.0890 (±0.0004)*
Δρ_M_ (×10^10^ cm g^−1^)	2.29
*R*_g_ (Å)	25.3 (±0.2)*
Experimental molecular mass (MM_exp_)^b^ (kDa)	22.0**±**0.6
Predicted molecular mass (MM_pr_) (kDa)	20.5
MM_exp_/MM_pr_	1.07

We use the convention for reporting SAXS data as outlined in ref [42].

a error is 1 S.D.

b MM_exp_  =  *I*(0)N_A_/[protein]Δρ_M_^2^.

* *I*(0) and *R*_g_ were derived from *P*(*r*) using GNOM.

The experimentally derived *P*(*r*) profile (Figure 2D) is characteristic of an elongated molecule [43], [44], and is consistent in overall dimensions (∼90×20 Å; *D_max_*  = 88 Å; *R_g_*  = 25.3 Å) with members of the NMR ensemble for Ldb1-Lhx3 (*R_g_* ^NMR^  = 24–25.1 Å; Figure 2A and B, Table S1). The 20 conformers of the NMR ensemble show a range of fits to the SAXS data, with *χ* ^2^ values ranging from 0.91 to 1.37 (Text S1, Table S1). The conformer that best fits the data is Model 17 (Figure 2C and D), which still shows a small deviation from the experimental SAXS data (e.g. Figure 2D).

To generate models of Ldb1-Lhx3 based solely on SAXS data, *ab initio* shape reconstructions of Ldb1-Lhx3, which are independent of high-resolution model bias, were calculated using DAMMIF. Each of the ten individual solutions used to generate the consensus DAMMIF shape of the complex (Figure 2E) fit the experimental data well (*χ* ^2^ of 0.7) and have an average normalised spatial discrepancy of 0.63 indicating that the solutions used to generate the consensus model are similar [39]. We also performed rigid-body modelling of Ldb1-Lhx3 using BUNCH [40] in which the positions of the two LIM-modules of the complex were allowed to flex relative to each other during refinement against the SAXS data, as described for related Lhx3/4-Isl1/2 complexes in [26]. The resultant BUNCH model fits the SAXS data with *χ* ^2^ = 0.75. Model 17 from the NMR ensemble, the BUNCH model and the DAMMIF shape reconstruction all superimpose well (Figure 2E). The differences between the BUNCH and NMR models are some twisting at the hinge/spacer, and repositioning of the unstructured linker, such that it is more compact in the BUNCH model. By definition, the positions of atoms in unstructured regions of the NMR ensemble are inadequately defined, so although the unstructured residues are present in each conformer their given coordinates are essentially arbitrary. Swapping linkers between models from the NMR ensemble to a more compact linker can improve the fit to the SAXS data, suggesting that inadequate modelling of the linker is a major contributor to the poor fits of some NMR conformers (Text S1, Figure S1, Table S2). Indeed, the structured regions (but not the undefined tethers) of the NMR ensemble superimpose well the DAMMIF consensus model (Figure 2F), indicating that the range of angles at the hinge/spacer in the NMR ensemble (Figure 2G) is reasonable.

Overall these data suggest that the NMR ensemble is a reasonable representation of the complex in solution: two relatively rigid LIM domains in an overall extended orientation with an interdomain angle that can vary by up to 30°.

### Analysis of Interactions between Lhx3 Mutants

**Figure 3:**
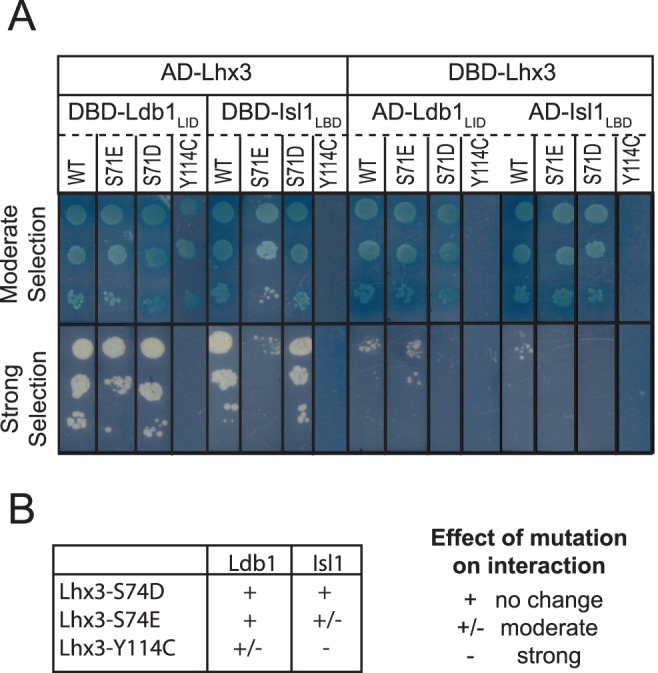
Effect of mutations in Lhx3 on interactions with Ldb1 and Isl1. Yeast two-hybrid data for Lhx3 (constructs comprised both LIM domains), the Lhx3 phosphomimic mutants Lhx3(S71D) and Lhx3(S71E) and the CPHDS mutation Lhx3(Y114C) against Ldb1_LID_ and Isl1_LBD_, and. Interaction data for both bait/prey orientations are shown; DBD and AD designate the plasmids pGBT9 and pGAD10, respectively. Serial dilutions of culture (A_600nm_  = 0.2, 0.02, and 0.002) were spotted onto each column of the plate with the highest concentration at the top. Two different selection conditions (moderate stringency  =  SD -L-W-H +3-AT; high stringency  =  SD-L-W-H-A). Data are representative of 2­ or 3 separate experiments. In all cases transformation control plates (SD-L-W) showed strong growth of yeast indicating successful transformations, and negative controls of each constructs versus the corresponding empty plasmid showed no yeast growth under the conditions tested.

We used yeast two-hybrid analysis to test if the Lhx3(Y114C) mutant and two pseudo phosphorylation mutants [45], Lhx3(S74D) and Lhx3(S74E), affected the interaction of that protein with Ldb1_LID_ or Isl1_LBD_ (Figure 3). In these experiments the apparent strength of the interactions depends on which vector contains the Lhx3 construct; yeast growth is more robust when Lhx3 is fused to the activation domain in the pGAD10 vector than to the DNA-binding domain in pGBT9 [13]. No interaction was observed between Lhx3(Y114C) and Isl1_LBD_ under any selection conditions, whereas yeast growth was observed under moderate (-L-W-H +3-AT) but not high stringency (-L-W-H-A) selection conditions for binding to Ldb1_LID_ when Lhx3 is in the pGAD10 vector, indicating that the mutation abrogates binding to Isl1_LBD_ and significantly reduces binding to Ldb1_LID_ (Figure 3). The Lhx3(S74D) phosphomimic did not appear to affect either interaction, whereas Lhx3(S74E) moderately reduced binding to Isl1_LBD_; no yeast growth was seen under high selection conditions (-L-W-H-A) when Lhx3 was in the pGAD10 vector.

### Characterisation of the Lhx3(Y114C) Mutant

**Figure 4:**
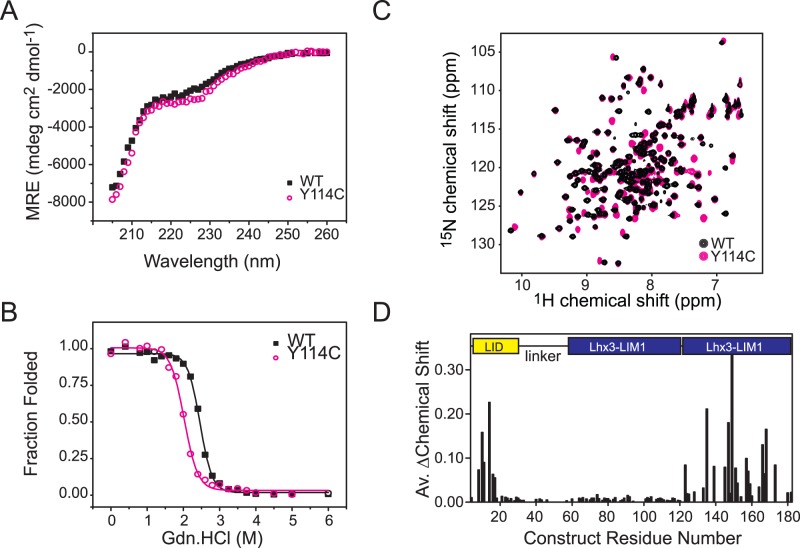
Effect of mutations on Lhx3. (**A**) Overlay of CD spectra from Ldb1-Lhx3 (*solid black squares*) and Ldb1-Lhx3(Y114C) (*open magenta circles*). Spectra were collected with 30 μM samples in 20 mM Na_2_HPO_4_, 40 mM NaCl, 1 mM DTT, pH 6.8 at 310K and were buffer baseline corrected. (**B**) Chemical denaturation of Ldb1-Lhx3 (*solid black squares*) and Ldb1-Lhx3(Y114C) (*open magenta circles*). Fraction folded was estimated using tryptophan fluorescence intensities (excitation wavelength 295 nm and emission wavelength 334 nm). Lines show the fits to a sigmoidal function. Proteins were at concentrations of 2.5 μM (**C**) ^15^N-HSQC spectra of Ldb1-Lhx3 (*black*, ∼800 μM)) and Ldb1-Lhx3(Y114C) (*magenta*, ∼200 μM) in 20 mM Na_2_HPO_4_, 40 mM NaCl, 1 mM DTT, pH 6.8 at 310K. (**D**) Analysis of chemical shift differences from panel C based on assignments for the wildtype protein [25] and inferred assignments for the mutant protein (Table S3). Peaks were identical for the C-terminal half of Ldb1_LID_, and Lhx3_LIM1_ which make direct contacts in the structure of the complex, but were significantly different for the N-terminal half of Ldb1_LID_, and Lhx3_LIM2._

A tethered Ldb1-Lhx3(Y114C) construct was generated to determine whether misfolding and/or non-native zinc ligation caused reduced binding by the Y114C mutant. Far-UV circular dichroism spectra of the wildtype and mutant proteins were very similar (Figure 4A) indicating that the wildtype and mutant proteins have identical levels of secondary structure (6–7% α-helical structure, 33–35% β-structure, and 58–61% coil). The relative stability of the mutant and wildtype tethered complexes was assessed using resistance to chemical denaturation monitored by intrinsic tryptophan fluorescence as described previously [13], [26], [46]. Both complexes contain a single tryptophan in Lhx3_LIM1_, and their tryptophan fluorescence emission spectra show a red-shift of wavelength maximum and reduction in intensity typical of unfolding when exposed to 6 M Gdn.HCl (data not shown). Both proteins displayed a monophasic unfolding transition (Figure 4B), but it should be noted that these proteins each contain two LIM domains and a binding peptide meaning that the folding was unlikely to be two-state or fully reversible and free energies of folding could not be determined. The mutant tethered complex (midpoint of denaturation, D_50%_  = 2.0 M Gdn.HCl) was apparently destabilised relative to the wildtype complex (D_50%_  = 2.5 M Gdn.HCl). The stability of these complexes stems from both the intrinsic stability of the LIM domains and the affinity of the LIM domains for Ldb1_LID_, and so the decrease in the overall apparent stability of the Ldb1_LID_-Lhx3(Y114C) mutant likely reflects a weaker interaction and/or decrease in stability of the Lhx3_LIM1+2._

The structure of the mutant protein was further probed by NMR spectroscopy. The peaks in the ^15^N-HSQC spectrum of uniformly labelled ^15^N-Ldb1-Lhx3(Y114C) were sharp and well dispersed indicating that the protein is folded; however, many of the peaks have shifted compared to the spectrum for the wildtype protein (Figure 4C); we have inferred assignments for the mutant protein from resonance proximity in two-dimensional spectra (Figure S2 and Table S3). Although caution should be taken in the interpretation of these spectra because we have not unambiguously assigned the spectra, the majority of the peaks that have moved in the spectrum of the mutant protein correspond to residues in the Lhx3_LIM2_ domain whereas the N-terminal half of Ldb1_LID_ and Lhx3_LIM1_ appear to be largely unaffected by the mutation (Figure 4D, Figure S2 and Table S3).

**Figure 5:**
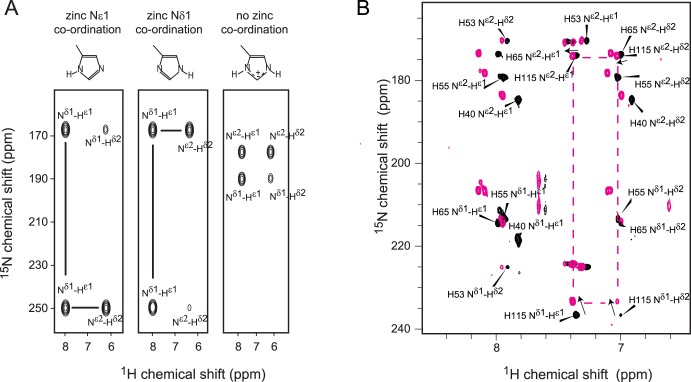
Ligation of zinc by histidine sidechains in Ldb1-Lhx3 is not affected by the Y114C mutation. (**A**) Schematic representation of chemical shift patterns arising from histidine protonation and expected zinc-ligation pattern adapted from ref [29]. (**B**) ^15^N-HSQC spectra showing histidine sidechains from wildtype Ldb1-Lhx3 (*black*, labelled) and mutant Ldb1-Lhx3(Y114C) (*magenta*)**.** Arrows indicate the chemical shift movement of H115.

We analysed the protonation pattern of the H115 sidechain using ^15^N-HSQC experiments [29] to investigate the possibility that the Y114C mutation might alter the zinc coordination. The two histidine residues that ligate zinc ions in Ldb1-Lhx3, Lhx3(H55) and Lhx3(H115), give rise to patterns that are typical of protonation at N^ε2^, indicating that zinc ligation occurs through N^δ1^ in both cases (Figure 5). Any change in the zinc coordination state of those histidine sidechains would result in changes to both the pattern and intensity of the peaks in the spectrum of the mutant protein (Figure 5A). Although there are some changes in the ^15^N chemical shift resonances from the N^δ1^ nitrogen of H115 in the spectrum from Ldb1-Lhx3(Y114C), the lack of movement of the resonances corresponding to the N^ε2^_,_ H^ε1^ and H^δ2^ nuclei suggests that the ligation state of H115 is the same in both the wildtype and mutant proteins (Figure 5B). That is, C114 in the mutant protein does not replace H115 as a zinc ligand. The movement of the N^δ1^ nitrogen of H115 can be attributed to its proximity to the site of mutation; a bulky aromatic tyrosine sidechain has been substituted by cysteine, likely causing a change in the local fold and/or electronic structure of the protein.

**Figure 6:**
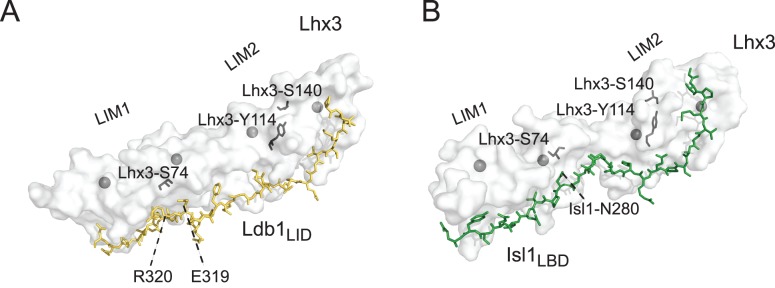
Potential roles of Lhx3-S74 and Lhx3-Y114. Views of murine Lhx3 (*white semi-transparent surfaces*) showing the position of S74, Y114 and S140 (*black sticks*). (**A**) Ldb1_LID_ (*yellow sticks*) and (**B**) Isl1_LBD_ (*green sticks*) are shown to indicate the positions of binding faces. Residues from Ldb1 and Isl1 that lie close to Lhx3-S74 are also shown. Zinc ions are shown as grey spheres. Model 17 from the NMR structure of Ldb1-Lhx3 (pdb accession code: 2JTN) and chain B from the X-ray structure of Lhx3-Isl1 (pdb accession code: 2RGT) were used to make these figures in Pymol.

## Discussion

The SAXS analysis of Ldb1-Lhx3 confirms that the previously published NMR structure of this tethered complex is a reasonable representation of its solution structure - an ensemble of conformers that varies in angle by up to ∼30° between the two LIM modules. Given the extended nature of this complex and the paucity of contacts between the two LIM modules we expect that the complex undergoes a limited amount of flexion at this point and/or twists as suggested by the BUNCH model (Figure 2). The SAXS data for Ldb1-Lhx3 are consistent with a small amount of flexion, but not with high levels of flexibility. Previously reported heteronuclear ^15^N-^1^H NOE values are consistent with no or limited flexion between the LIM domains, although increased motion in residues Ldb1(E313–G315) corresponds with a short region of disorder in Ldb1_LID_ spacer [13]. Flexibility at the hinge/spacer was suggested for a related LIM-only protein 2 (Lmo2)-Ldb1_LID_ complex [47], and is consistent with SAXS data for Lhx3/4-Isl1/2 complexes [26]. A phenylalanine residue followed by a glycine residue is highly conserved at this hinge in LIM-homeodomain and LIM-only proteins (Figure S3), suggesting that some flexion at the hinge/spacer is a common phenomenon in this type of complex. Flexibility was suggested to play a role in the binding of Lmo2/Ldb1 to Tal1/E47 [47], and differences in the inter-LIM domain angle could influence the recruitment of additional binding partners to and thereby generate differences in activity between alternate Lhx3-containing complexes. We recently showed that the complex formed between LIM homeobox protein 4 (Lhx4) and Islet 2 (Isl2) and that formed between Lhx3 and Isl2 have a more compact average structure (*D_max_* ∼75 Å) than a similar complex formed by Lhx3 and Isl1 (*D_max_* ∼90 Å), suggesting that the binding partner can influence the average angle formed between the two LIM domains [26]. Despite a higher sequence identity between Isl1_LBD_ and Isl2_LBD_ than Isl1_LBD_ and Ldb1_LID_, SAXS data for Ldb1-Lhx3 (which show, for example, a maximum dimension *D_max_* of 88 Å) indicate that the gross structure of this complex resembles the more elongated Lhx3/4-Isl1 complexes. The spacing and structure of spacer may play an important role in determining the inter-LIM angle; the spacers in Isl1_LBD_ and Isl2_LBD_ have very low sequence identity compared to the LIM-binding motifs in those domains.

Although S74 lies proximal to the binding faces in both complexes (Figure 6), neither of the phosphomimic mutants of Lhx3 had a major effect on binding to Ldb1 and Isl1 using yeast two-hybrid assays. Only S74E showed some apparent reduction in binding suggesting that the larger size (rather than the charge) of the glutamate sidechain was responsible for this effect. It is plausible that in Lhx3(S74E) local rearrangements in the LIM1 domain may be required to accommodate the larger glutamate sidechain resulting in a minor reduction of binding. These results are consistent with data from Parker et al. showing that Lhx3(S74A) mutants did not affect binding of Lhx3 to Ldb1, PIT-1 or MRG in GST-pulldown experiments [10]. If Lhx3 is phosphorylated at this site as part of normal activity it may be to modulate binding to as yet unidentified or uncharacterised protein partners.

Our yeast two-hybrid data using the CPHDS-associated mutation Lhx3(Y114C) confirm the reduced binding observed between Lhx3 mutants and Ldb1 in GST-pulldown experiments [6]. That the mutation also severely affects interactions with Isl1 is consistent with our structural and mutagenic scanning data showing that Isl1 and Ldb1 interact with the same site on Lhx3, with the majority of critical contacts being made at the LIM2 domain [13]. Y114 is highly conserved in the LIM domains of LIM-homeodomain and LIM-only proteins (Figure S3) suggesting that Y114 is crucial for the structure and/or function of these proteins. Y114 does not contact either Ldb1_LID_ or Isl1_LBD_ (Figure 6A), nor does the Lhx3(Y114C) mutation appear to induce a change in zinc ligation (Figure 5). Rather, the highly buried sidechain points away from the Ldb1_LID_ and Isl1_LBD_ binding interfaces and forms a major part of the hydrophobic core of the first zinc binding module in Lhx3_LIM2_. Substitution of the bulky tyrosine sidechain with cysteine could result in the repacking of the hydrophobic core of Lhx3_LIM2_, which is consistent with the major chemical shift changes observed in the NMR spectrum of the Ldb1-Lhx3(Y114C) mutant and the reduced stability of Ldb1-Lhx3. However, Lhx3(Y114F) has a similar phenotype to Lhx3(Y114C) [5] suggesting that other factors may be at play. The hydroxyl group in the Y114 sidechain forms a hydrogen bond with the main-chain carbonyl from Lhx3(S140) (Figure 6), which would be lost on both mutants potentially destabilising the domains. Although interactions between Lhx3 and Ldb1 and Isl1 are required for motor neuron development [2], patients carrying the mutation do not show signs of motor neuron impairment [4]. It is likely that the closely related Lhx4 protein, which is also expressed in motor neurons [3], may compensate for mutant Lhx3 in developing motor neurons but not in pituitary development [48]. In motor neurons both *Lhx3* and *Lhx4* have high levels of expression, whereas only Lhx3 is highly expressed at multiple stages of development, but Lhx4 appears to have a much more restricted expression pattern (MGI database, http://www.informatics.jax.org/).

In conclusion, our analysis of Lhx3 complexes provides a structural framework with which to understand how Lhx3 is regulated in the cell, and how the biological functions of the protein may be affected by disease causing mutations.

## Supporting Information

Figure S1 **Analysis of SAXS data.** (**A**) Models 1 (cyan linker), 8 (magenta linker), 9 (orange linker) and 17 (black linker) from the NMR structure of Ldb1-Lhx3 (pdb accession code: 2JTN) with Ldb1_LID_ in yellow and Lhx3 in blue; zinc ions are depicted as grey spheres. The models were aligned over the backbone atoms of Lhx3_LIM1_ and the corresponding region of Ldb1. (**B**) Scattering data for Ldb1-Lhx3 (*grey circles*) shown as *I*(*q*) against *q* plots. Curves are the fits to the data generated by CRYSOL of sample NMR models from the Ldb1-Lhx3 NMR ensemble as indicated. Note that the scale on the Y-axis is arbitrary and the data/fits have been plotted separately for clarity.
(EPS)

Figure S2 **Overlay of ^15^N-HSQC spectra for wildtype and mutant Ldb1-Lhx3 constructs.** ^15^N-HSQC spectra of Ldb1-Lhx3 (black; 800 μM protein) and Ldb1-Lhx3(Y114C) (red; ∼200 μM protein). Spectra were collected at 310 K in 20 mM Na_2_HPO_4_, 40 mM NaCl, 1 mM DTT, pH 6.8. Assignments for wildtype Ldb1-Lhx3 [25] are shown in black; for clarity, not all assignments are shown. Note that construct residues 1–45 refer to Ldb1_295–339_, construct residues 46–56 correspond to the synthetic linker, and construct residues 57–182 refer to Lhx3_28–153_ (See Table S3 for full list).
(EPS)

Figure S3 **Sequence alignment of LIM domains from murine LIM-HD and LMO proteins.** Zinc-coordinating residues are marked with an asterisk. Gaps in the sequence are indicated with a dash. Residues equivalent murine Lhx3_Y114_ are highlighted in yellow. Residues equivalent to LMO2_F88/G89_ are highlighted in cyan and Lhx3_S74_ is highlighted in green. Consensus sequences are indicated in red. Φ, hydrophobic; +, positively charged; –, negatively charged; Z, aspartate or histidine.
(EPS)

Table S1 **Fits to the SAXS data for the individual models from the NMR models**. The fits are reported as χ^2^ values. For the top section of the table, each of the NMR models was fitted as the intact molecule, or with the unstructured tether and tail regions removed.
(DOCX)

Table S2 **Fits to the SAXS data for “swapped” tethers.** The chimeras were generated by swapping the linkers between the indicated models. Value reported are χ^2^ values of the fit.
(DOCX)

Table S3 **Inferred assignments of resonances in the ^15^N-HSQC of Ldb1-Lhx3(Y114C).** Values are given for the wildtype assignments [25], the inferred assignments for the Y114C mutant and the weighted average chemical shift differences between those assignments. Note that not all peaks could be assigned by this approach. The shaded parts of the table refer to assignments in the N-terminal half of Ldb1_LID_ and Lhx3_LIM2._
(DOCX)

Text S1 **Fits of NMR structures and SAXS data.**
(DOCX)
