# Supplementary material for: Solution Structure of the LIM-Homeodomain Transcription Factor Complex Lhx3/Ldb1 and the Effects of a Pituitary Mutation on Key Lhx3 Interactions
Source: PLoS One. 2012 Jul 25;7(7):e40719. doi: 10.1371/journal.pone.0040719 (PMC3405102; doi:10.1371/journal.pone.0040719)
Supplement: Table S1 — Fits to the SAXS data for the individual models from the NMR models. The fits are reported as χ2 values. For the top section of the table, each of the NMR models was fitted as the intact molecule, or with the unstructured tether and tail regions removed. (DOCX) [file pone.0040719.s004.docx]

**Table S1.** Fits to the SAXS data for the individual models from the NMR models. The fits are reported as χ^2^ values. For the top section of the table, each of the NMR models was fitted as the intact molecule, or with the unstructured tether and tail regions removed.

| **Complete models** | | | **Structured regions*** | | |
| --- | --- | --- | --- | --- | --- |
| **Model** | χ^2^ | ***R*_g_** | **Model** | χ^2^ | ***R*_g_** |
| 1 | 1.26 | 25.1 | 1 | 1.10 | 22.1 |
| 2 | 1.19 | 24.5 | 2 | 1.29 | 21.8 |
| 3 | 1.08 | 24.9 | 3 | 1.16 | 22.0 |
| 4 | 1.32 | 24.7 | 4 | 1.29 | 21.7 |
| 5 | 1.15 | 24.6 | 5 | 1.45 | 21.6 |
| 6 | 1.25 | 24.8 | 6 | 1.26 | 21.9 |
| 7 | 1.17 | 24.8 | 7 | 0.99 | 22.2 |
| 8 | 0.97 | 24.0 | 8 | 1.38 | 21.7 |
| 9 | 1.37 | 24.7 | 9 | 1.28 | 21.8 |
| 10 | 1.24 | 24.9 | 10 | 0.97 | 22.3 |
| 11 | 1.21 | 24.9 | 11 | 1.13 | 22.0 |
| 12 | 0.93 | 24.4 | 12 | 1.27 | 21.8 |
| 13 | 1.29 | 24.9 | 13 | 1.23 | 21.9 |
| 14 | 1.13 | 24.5 | 14 | 1.16 | 22.0 |
| 15 | 1.23 | 24.9 | 15 | 1.20 | 21.9 |
| 16 | 1.19 | 24.8 | 16 | 1.16 | 22.0 |
| 17 | 0.91 | 24.4 | 17 | 1.28 | 21.8 |
| 18 | 1.24 | 24.8 | 18 | 1.01 | 22.2 |
| 19 | 1.27 | 25.0 | 19 | 1.10 | 22.1 |
| 20 | 1.13 | 24.6 | 20 | 1.31 | 21.7 |

*Residues 1-6 (N-terminal region of Ldb1_LID_), 34-56 (C-terminal region of Ldb1_LID_, glycine/serine linker and N-terminal residues from Lhx3_LIM1+2_) and 181-182 (C-terminal residues from Lhx3_LIM1+2_) from the constructs used in the NMR ensemble were omitted.
