# Supplementary material for: Solution Structure of the LIM-Homeodomain Transcription Factor Complex Lhx3/Ldb1 and the Effects of a Pituitary Mutation on Key Lhx3 Interactions
Source: PLoS One. 2012 Jul 25;7(7):e40719. doi: 10.1371/journal.pone.0040719 (PMC3405102; doi:10.1371/journal.pone.0040719)
Supplement: Table S2 — Fits to the SAXS data for “swapped” tethers. The chimeras were generated by swapping the linkers between the indicated models. Value reported are χ2 values of the fit. (DOCX) [file pone.0040719.s005.docx]

**Table S2. Fits to the SAXS data for “swapped” tethers.** The chimeras were generated by swapping the linkers between the indicated models. Value reported are χ^2^ values of the fit.

|  | **Tether 1** | **Tether 8** | **Tether 17** |
| --- | --- | --- | --- |
| Model 1 | 1.26 | 1.05 | 1.33 |
| Model 8 | 1.16 | 0.97 | 1.23 |
| Model 17 | 0.89 | 0.82 | 0.91 |
