# Supplementary material for: Solution Structure of the LIM-Homeodomain Transcription Factor Complex Lhx3/Ldb1 and the Effects of a Pituitary Mutation on Key Lhx3 Interactions
Source: PLoS One. 2012 Jul 25;7(7):e40719. doi: 10.1371/journal.pone.0040719 (PMC3405102; doi:10.1371/journal.pone.0040719)
Supplement: Table S3 — Inferred assignments of resonances in the 15N-HSQC of Ldb1-Lhx3(Y114C). Values are given for the wildtype assignments [25], the inferred assignments for the Y114C mutant and the weighted average chemical shift differences between those assignments. Note that not all peaks could be assigned by this approach. The shaded parts of the table refer to assignments in the N-terminal half of Ldb1LID and Lhx3LIM2. (DOCX) [file pone.0040719.s006.docx]

**Table S3: Inferred assignments of resonances in the ^15^N-HSQC of Ldb1-Lhx3(Y114C).** Values are given for the wildtype assignments [[26](#_ENREF_26)], the inferred assignments for the Y114C mutant and the weighted average chemical shift differences between those assignments. Note that not all peaks could be assigned by this approach. The shaded parts of the table refer to assignments in the N-terminal half of Ldb1_LID_ and Lhx3_LIM2._

| **Residue Number** | | **Wildtype** | | **Y114C** | |  |
| --- | --- | --- | --- | --- | --- | --- |
| Ldb1-Lhx3  Construct | Protein Sequence | N  (ppm) | HN (ppm) | N  (ppm) | HN (ppm) | Weighted Average  Chemical Shift Difference (ppm) |
| 4 | Ldb1-298 | 120.796 | 7.899 | 120.739 | 7.904 | 0.010 |
| 8 | 302 | 126.637 | 8.266 | 126.661 | 8.339 | 0.073 |
| 10 | 304 | 128.055 | 9.139 | 128.954 | 9.215 | 0.158 |
| 11 | 305 | 118.136 | 8.906 | 118.688 | 8.936 | 0.090 |
| 14 | 308 | 113.964 | 8.609 | 112.667 | 8.503 | 0.226 |
| 16 | 310 | 125.31 | 8.768 | 125.513 | 8.823 | 0.063 |
| 17 | 311 | 105.647 | 8.6 | 105.759 | 8.548 | 0.055 |
| 18 | 312 | 107.253 | 8.13 | 107.202 | 8.126 | 0.009 |
| 21 | 315 | 111.107 | 8.623 | 111.086 | 8.61 | 0.013 |
| 23 | 317 | 118.175 | 8.851 | 118.134 | 8.842 | 0.011 |
| 24 | 318 | 118.858 | 7.854 | 118.8 | 7.853 | 0.009 |
| 25 | 319 | 120.981 | 7.292 | 120.981 | 7.289 | 0.003 |
| 26 | 320 | 126.077 | 8.796 | 126.028 | 8.795 | 0.008 |
| 28 | 322 | 123.721 | 7.653 | 123.705 | 7.654 | 0.003 |
| 29 | 323 | 123.047 | 8.859 | 122.979 | 8.862 | 0.011 |
| 30 | 324 | 126.09 | 8.722 | 126.03 | 8.721 | 0.009 |
| 31 | 325 | 127.936 | 9.423 | 127.906 | 9.424 | 0.005 |
| 32 | 326 | 122.82 | 8.725 | 122.801 | 8.725 | 0.003 |
| 33 | 327 | 124.194 | 8.747 | 124.168 | 8.747 | 0.004 |
| 40 | 334 | 116.843 | 8.035 | 116.806 | 8.033 | 0.006 |
| 42 | 336 | 119.71 | 7.909 | 119.662 | 7.909 | 0.007 |
| 43 | 337 | 123.975 | 8.323 | 123.949 | 8.326 | 0.005 |
| 46 | Linker-1 | 109.411 | 8.433 | 109.377 | 8.433 | 0.005 |
| 57 | Lhx3-28 | 115.901 | 7.99 | 115.897 | 7.987 | 0.003 |
| 60 | 31 | 125.223 | 8.121 | 125.179 | 8.124 | 0.007 |
| 64 | 35 | 132.468 | 8.517 | 132.413 | 8.513 | 0.009 |
| 65 | 36 | 112.594 | 9.484 | 112.555 | 9.487 | 0.007 |
| 66 | 37 | 118.163 | 7.745 | 118.104 | 7.741 | 0.010 |
| 68 | 39 | 114.627 | 7.736 | 114.588 | 7.733 | 0.007 |
| 70 | 41 | 120.835 | 9.81 | 120.802 | 9.812 | 0.005 |
| 71 | 42 | 127.746 | 8.867 | 127.687 | 8.867 | 0.009 |
| 72 | 43 | 118.54 | 6.754 | 118.489 | 6.749 | 0.009 |
| 74 | 45 | 112.915 | 7.598 | 112.808 | 7.597 | 0.017 |
| 75 | 46 | 120.782 | 9.175 | 120.743 | 9.169 | 0.008 |
| 77 | 48 | 123.816 | 9.325 | 123.79 | 9.323 | 0.004 |
| 78 | 49 | 126.436 | 8.891 | 126.379 | 8.893 | 0.009 |
| 79 | 50 | 123.253 | 10.016 | 123.199 | 10.016 | 0.008 |
| 80 | 51 | 110.975 | 8.763 | 110.928 | 8.761 | 0.008 |
| 81 | 52 | 119.118 | 7.812 | 119.104 | 7.809 | 0.004 |
| 83 | 54 | 117.19 | 9.062 | 117.154 | 9.062 | 0.006 |
| 84 | 55 | 121.183 | 9.262 | 121.129 | 9.256 | 0.010 |
| 87 | 58 | 117.034 | 7.543 | 117.019 | 7.544 | 0.003 |
| 88 | 59 | 124.141 | 7.031 | 124.107 | 7.03 | 0.005 |
| 89 | 60 | 122.635 | 7.278 | 122.578 | 7.276 | 0.009 |
| 91 | 62 | 126.455 | 9.143 | 126.369 | 9.137 | 0.015 |
| 92 | 63 | 121.378 | 8.845 | 121.303 | 8.846 | 0.012 |
| 94 | 65 | 115.824 | 7.655 | 115.838 | 7.656 | 0.002 |
| 101 | 72 | 115.39 | 8.838 | 115.345 | 8.837 | 0.007 |
| 102 | 73 | 117.663 | 8.598 | 117.609 | 8.593 | 0.010 |
| 103 | 74 | 115.485 | 8.674 | 115.448 | 8.669 | 0.008 |
| 104 | 75 | 124.11 | 8.158 | 124.035 | 8.16 | 0.012 |
| 107 | 78 | 115.881 | 7.8 | 115.842 | 7.798 | 0.006 |
| 108 | 79 | 118.975 | 7.955 | 118.923 | 7.955 | 0.008 |
| 109 | 80 | 120.472 | 9.075 | 120.414 | 9.077 | 0.009 |
| 110 | 81 | 119.374 | 9.452 | 119.3321 | 9.45 | 0.007 |
| 111 | 82 | 121.015 | 8.73 | 120.991 | 8.733 | 0.005 |
| 113 | 84 | 120.767 | 8.875 | 120.756 | 8.879 | 0.004 |
| 114 | 85 | 121.625 | 8.8 | 121.607 | 8.801 | 0.003 |
| 115 | 86 | 115.556 | 7.944 | 115.568 | 7.942 | 0.003 |
| 116 | 87 | 120.15 | 7.846 | 120.116 | 7.839 | 0.009 |
| 117 | 88 | 116.113 | 7.2474 | 116.086 | 7.24 | 0.008 |
| 119 | 90 | 108.317 | 8.015 | 108.296 | 8.017 | 0.004 |
| 120 | 91 | 119.701 | 8.879 | 119.687 | 8.877 | 0.003 |
| 123 | 94 | 132.107 | 8.804 | 132.312 | 8.726 | 0.084 |
| 124 | 95 | 120.546 | 8.642 | 120.466 | 8.656 | 0.019 |
| 125 | 96 | 116.813 | 8.135 | 116.792 | 8.117 | 0.018 |
| 128 | 99 | 108.351 | 8.351 | 108.309 | 8.373 | 0.023 |
| 133 | 104 | 122.193 | 7.379 | 122.371 | 7.396 | 0.032 |
| 135 | 106 | 117.853 | 9.124 | 116.616 | 9.034 | 0.211 |
| 139 | 110 | 121.609 | 9.059 | 121.624 | 9.14 | 0.081 |
| 145 | 116 | 123.104 | 8.994 | 122.651 | 9.031 | 0.079 |
| 147 | 118 | 119.112 | 6.76 | 120.066 | 6.864 | 0.180 |
| 148 | 119 | 124.737 | 7.272 | 124.839 | 7.284 | 0.020 |
| 149 | 120 | 126.588 | 7.2 | 125.966 | 6.86 | 0.353 |
| 151 | 122 | 126.443 | 9.264 | 126.939 | 9.257 | 0.077 |
| 152 | 123 | 125.497 | 9.27 | 125.462 | 9.227 | 0.043 |
| 154 | 125 | 116.12 | 7.557 | 116.079 | 7.547 | 0.012 |
| 157 | 128 | 128.447 | 8.449 | 128.952 | 8.387 | 0.099 |
| 158 | 129 | 125.889 | 9.043 | 126.106 | 8.981 | 0.070 |
| 159 | 130 | 115.728 | 8.124 | 115.756 | 8.165 | 0.041 |
| 160 | 131 | 116.454 | 9.148 | 116.412 | 9.16 | 0.014 |
| 164 | 135 | 116.322 | 9.267 | 116.466 | 9.293 | 0.034 |
| 166 | 137 | 123.466 | 8.95 | 124.267 | 8.991 | 0.130 |
| 167 | 138 | 120.635 | 9.027 | 120.499 | 8.966 | 0.064 |
| 168 | 139 | 117.448 | 7.875 | 116.499 | 7.951 | 0.165 |
| 173 | 144 | 127.746 | 10.114 | 128.172 | 10.167 | 0.084 |
| 180 | 151 | 124.502 | 7.734 | 124.635 | 7.729 | 0.021 |
| 182 | 153 | 126.148 | 7.656 | 126.18 | 7.677 | 0.022 |
