# Supplementary material for: Solution Structure of the LIM-Homeodomain Transcription Factor Complex Lhx3/Ldb1 and the Effects of a Pituitary Mutation on Key Lhx3 Interactions
Source: PLoS One. 2012 Jul 25;7(7):e40719. doi: 10.1371/journal.pone.0040719 (PMC3405102; doi:10.1371/journal.pone.0040719)
Supplement: Text S1 — Fits of NMR structures and SAXS data. (DOCX) [file pone.0040719.s007.docx]

**Text S1: Fits of NMR structures and SAXS data.**

To explore the contribution of the unstructured regions of the protein to the SAXS data, theoretical scattering profiles of the 20 conformers from the NMR ensemble were determined both for the intact models and for models lacking the unstructured regions (the glycine/serine tether and short stretches at the ends of the construct (Figure S1, Table S1). For the intact models fits ranged from χ^2^ = 0.92–1.37. Overall, removing the tether resulted in a slightly poorer fit to the data (χ^2^ = 1.0–1.5), which can be accounted for by the removal of mass. To crudely mimic sampling of multiple conformations by the unstructured regions theoretical scattering profiles were also calculated for some models in which the tethers were swapped (Models 1, 8 and 17 in the NMR ensemble; by eye the tethers in these models appear the most divergent; Figure S1A) and fitted to the experimental scattering data (Table S2). The Model 8 tether resulted in the best fit in all cases. *Ab initio* shape restoration from SAXS data using DAMMIF, and rigid body refinement using BUNCH (Figure 2) indicate that the tether likely adopts a more compact structure than that suggested by the NMR structures, which is line with observations that the most compact linker (from Model 8) gave the best fits to the SAXS data for all models tested. We note that by definition the unstructured regions in the NMR ensemble are not adequately modelled, but that removing the tethers results in worse fits to the scattering data than retaining them. This simple analysis indicates that the flexible tethers in this type of complex do make a contribution to the SAXS data, and fits of high resolution structures generated by standard NMR or X-ray methods (which do not provide information about disordered regions) to such data are likely be slightly compromised by this phenomenon.
